# Intramedullary Nail for Treatment of Proximal Humeral Fracture: A Credible Fixation in Comminuted Calcar

**DOI:** 10.1111/os.13616

**Published:** 2022-12-13

**Authors:** Huichao Fu, Jianhong Wu, Xiaoming Wu

**Affiliations:** ^1^ Department of Orthopaedic Traumatology, Trauma Center, Shanghai General Hospital Shanghai China

**Keywords:** Calcar Comminution, Intramedullary Nail, Neck Shaft Angle, Proximal Humeral Fracture

## Abstract

**Objective:**

Restoration of the medial support is especially important for the treatment of proximal humeral fractures. The objective of this study was to investigate the radiographic and clinical outcomes of intramedullary nail fixation with a special focus on the presence of calcar comminution.

**Methods:**

In this retrospective study of patients with displaced proximal humeral fractures that were treated by intramedullary nail between January 2018 and July 2021, fracture morphology and the calcar integrity were noted on preoperative radiographs. Patients were divided into two groups according to calcar integrity. During follow‐up, radiological assessment and functional outcome, including the deltoid tuberosity index (DTI), neck shaft angle (NSA), visual analog scale (VAS), the American Shoulder and Elbow Surgeons (ASES) score, the Simple Shoulder Test (SST) score, active and passive range of motion, were performed. A Student t‐test and univariate logistic regression analysis was used.

**Results:**

A total of 83 patients (54 female, 29 male) had complete follow‐up (average, 12.8 months; range, 10 to 33 months) and functional assessment in our study. The average age was 58.6 years (range, 20 to 89 years). The mean loss of NSA was 4° (range, 0°–12°) and no significant difference was found between two groups (*p* = 0.27). DTI had an average of 1.50 ± 0.19 (range 1.13–2.04). Patients with intact calcar achieved greater range of forward elevation (129.06 ± 11.91 vs. 121.05 ± 11.97, *p* = 0.01), and higher SST scores (8.61 ± 1.85 vs. 7.37 ± 2.22, *p* = 0.02). Two groups showed similar outcomes in VAS, ASES score, and range of abduction. One patient demonstrated a proximal interlocking screw cutting through and osteonecrosis of the humeral head, who underwent a second surgery for screw removal. There were no cases of infection, malunion, nonunion, nerve injury, subacromial impingement, or rotator cuff tear during the study period.

**Conclusion:**

Intramedullary nail can favorably be used to manage proximal humeral fractures with good early radiographic and functional outcomes, even for those with comminuted calcar.

## Graphical abstract

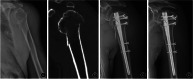

## Introduction

Proximal humerus fracture (PHF) is the third most common fracture in adults, accounting for 4% to 10% of all fractures.^1^ Due to the aging of population and its associated bone quality impairment, the incidence of comminuted and unstable PHF is increasing.^2^ More than 70% occurred in patients over 60 years old,^3^ and they are 3–4 times more common in elderly women than men.^1^, ^4^

While most PHFs are minimally displaced or non‐displaced, for which conservative management is usually recommended,^1^, ^5^ controversy exists regarding optimal management of displaced and comminuted PHF.^6^, ^7^ There are a number of fixation techniques, such as tension‐band routing, locking plate fixation, and intramedullary nailing over the past decade^8^, ^9^, ^10^, ^11^, ^12^, ^13^, ^14^; however, no single technique demonstrated evidence‐based superiority. Although locking plate fixation is considered as the standard treatment, complication rates are still unsatisfactory.^15^, ^16^, ^17^, ^18^, ^19^, ^20^ One of the major complications of surgical management in the elderly is secondary varus displacement associated with fixation failure due to screw cut‐out or cut‐through.^21^

Recent studies have discussed that the restoration of medial support has an important impact on clinical outcome and it is accomplished by cortical anatomical reduction, or placement of calcar screws.^22^, ^23^, ^24^ Whereas, for comminuted fractures in the calcar area, these approaches may be technically demanding and may even fail. Russo *et al*.^25^ created a fracture classification system based on the theory of controlled volume that can aid surgeons in rapid assessment. The involved fracture table was generated from an assessment of the condition of the calcar. Osterhoff et al.^26^ investigated the effect of calcar comminution on radiographic and clinical outcomes following locking plate fixation and found calcar comminution to be a correlated and readily detectable prognostic predictor for functional and subjective outcomes of these fractures.

Up to date, the application of intramedullary nailing in medial calcar comminuted proximal humerus fractures remains unknown. The use of nails to treat comminuted metaphyseal fractures may be suspicious; however, compared to plating, the proximal humeral nail has several theoretical advantages, such as minimal soft tissue damage and preservation of the blood supply to the already injured humeral head. Furthermore, centrally placed nails possess biomechanical advantages in resisting loss of reduction and varus displacement.^27^

In our trauma center, an angle‐stable, straight, short humerus nail (TRIGEN Humeral Nail; Smith & Nephew, Cordova, TN, USA) is commonly utilized for PHFs. This study aims to investigate (1) the radiographic results after intramedullary nail fixation, (2) evaluate the clinical outcomes, (3) and focus on the impact of medial calcar integrity.

## Materials and Methods

### Study Group

**Fig. 1:**
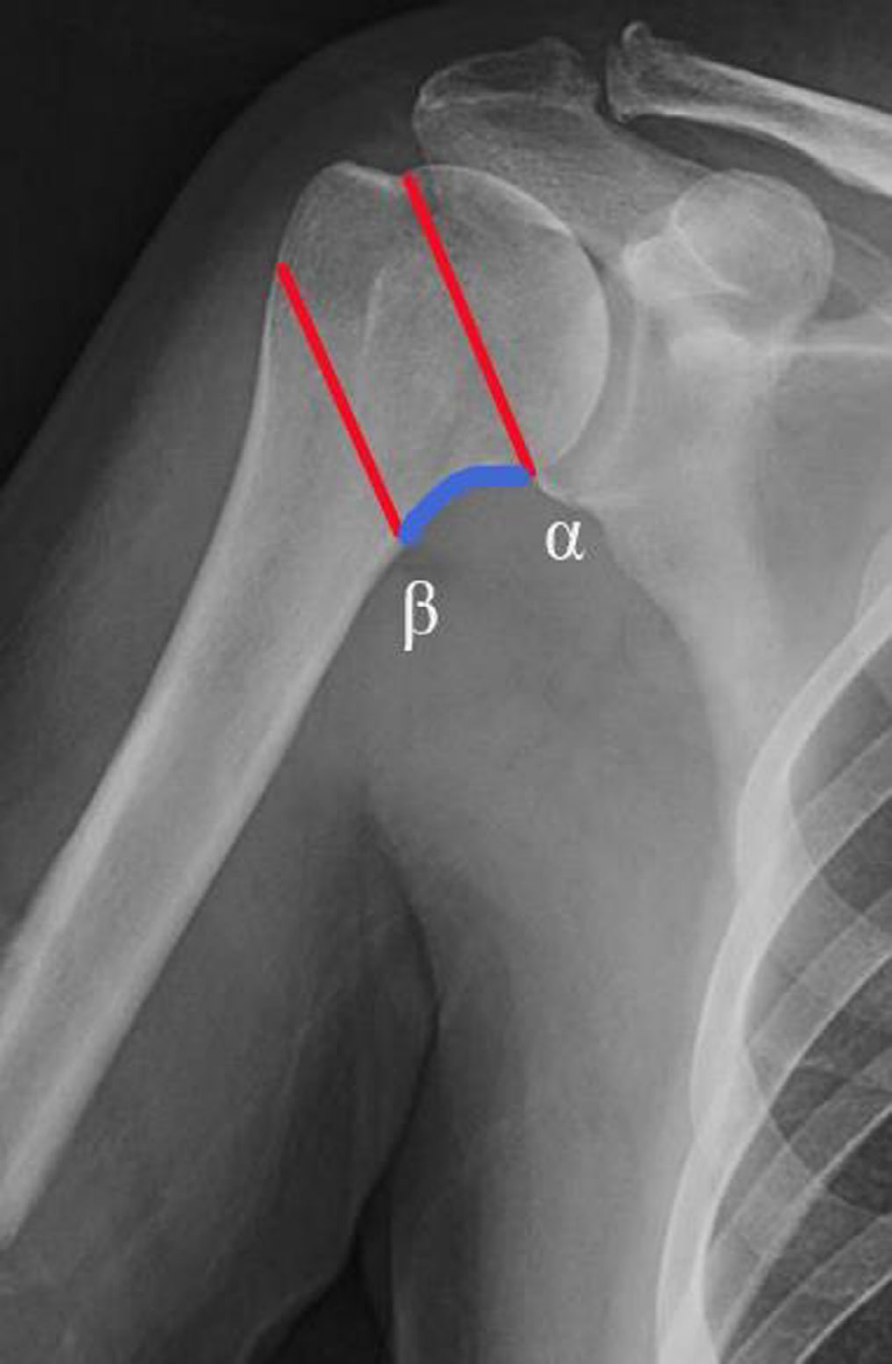
α plane: anatomical humeral neck plane; β plane: surgical neck plane. Calcar area is outlined in blue as the fifth fragment

In a single‐center study approved by the Institutional Review Board of the Hospital (2022SQ218), 83 patients with displaced proximal humeral fractures treated with a proximal humeral nail (TRIGEN Humeral Nail; Smith & Nephew, Cordova, TN, USA) between January 2018 and July 2021 were included cumulatively. Inclusion criteria were fresh closed two‐ to four‐part displaced proximal humeral fractures according to NEER classification^28^ in patients with an age more than 18 years and who were autonomous prior to fracture. The minimum follow‐up was 10 months. Exclusion criteria for the study were patients with disorders of the neuromuscular system, pathological fractures, open fractures, fractures with previous shoulder pathology, and those whose narrow humeral medullary cavity could not accommodate intramedullary nailing. Patients were divided into two groups. Group A consisted of patients with intact calcar. Group B consisted of patients with comminuted calcar, of which calcar fractured on both α and β planes (Figure 1).

### Operative Technique

**Fig. 2:**
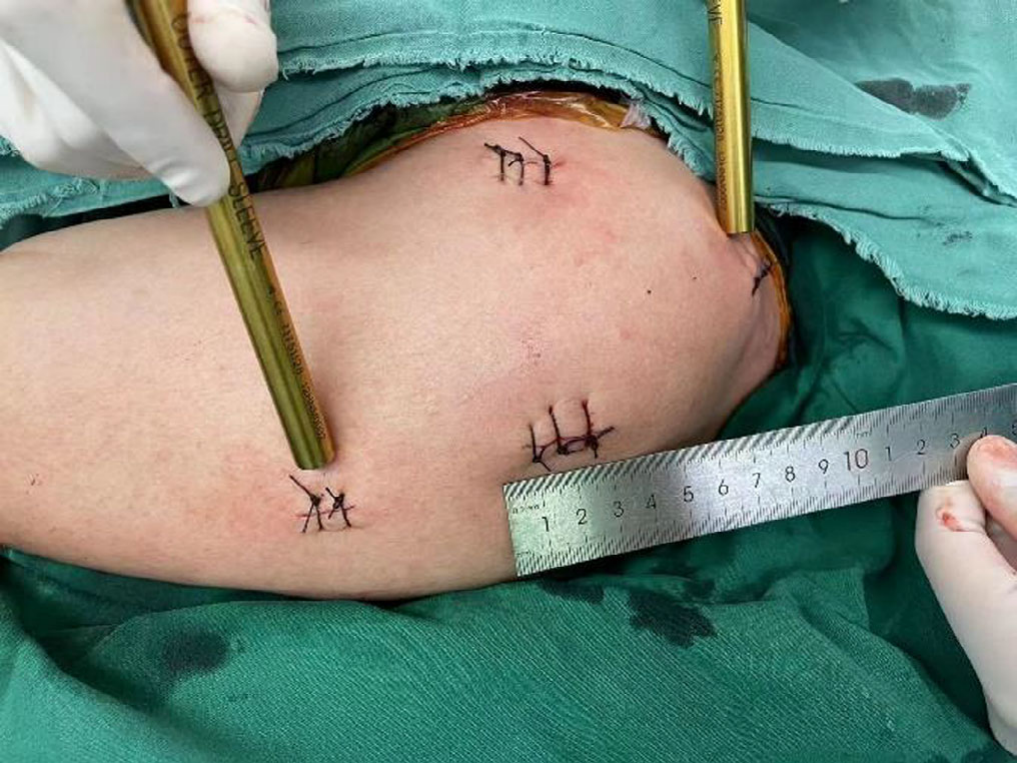
Minimal incisions of surgery

All surgical procedures were performed by one of the three senior shoulder surgeons (W. X., W. J., F. H.). Patients had their surgery under general anesthesia, placed in a beach chair position with the forearm supported by an arm rest. In cases where closed reduction was possible, nail insertion was performed percutaneously under fluoroscopic control. To correctly position the entry point, the humeral head were reduced with the aid of K‐wires as a joystick. The entry point was located at the apex of the humeral head and was aligned with the central line of the medullary canal in both anterior and lateral views. After correctly finding the entry point and penetrating the guide wire under fluoroscopic guidance through the fracture site into the humeral medullary cavity, the straight nail was inserted. If the reduction was not achieved by closed maneuvers, an additional incision was performed. The minimal displaced or non‐displaced tuberosity fragments were then reduced anatomically and fixed with proximal interlocking screws. In case of tuberosity comminution or osteoporotic bone, suture technique was used if necessary. Two distal locking screws were inserted through a targeting sleeve. All surgical steps were minimally invasive (Figure 2).

Postoperatively, a sling was used to protect the operated limb and was removed progressively. Rehabilitation exercises, such as pendular movement, progressive passive and active range of motion exercises, were started from the beginning.

### Radiographic and Functional Evaluation

**Fig. 3:**
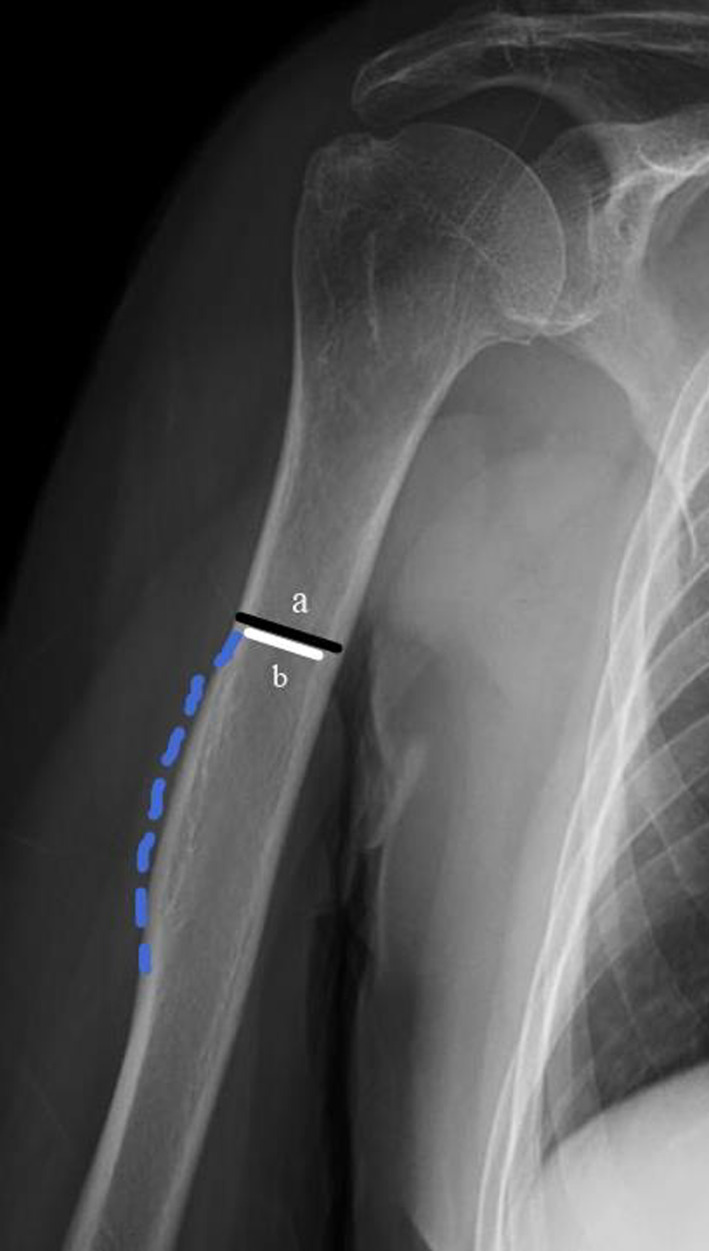
The deltoid tuberosity index is measured directly proximal to the deltoid tuberosity, where the outer cortical borders become parallel. At this level, the ratio between the outer cortical and the inner endosteal diameter is calculated (a/b)

**Fig. 4:**
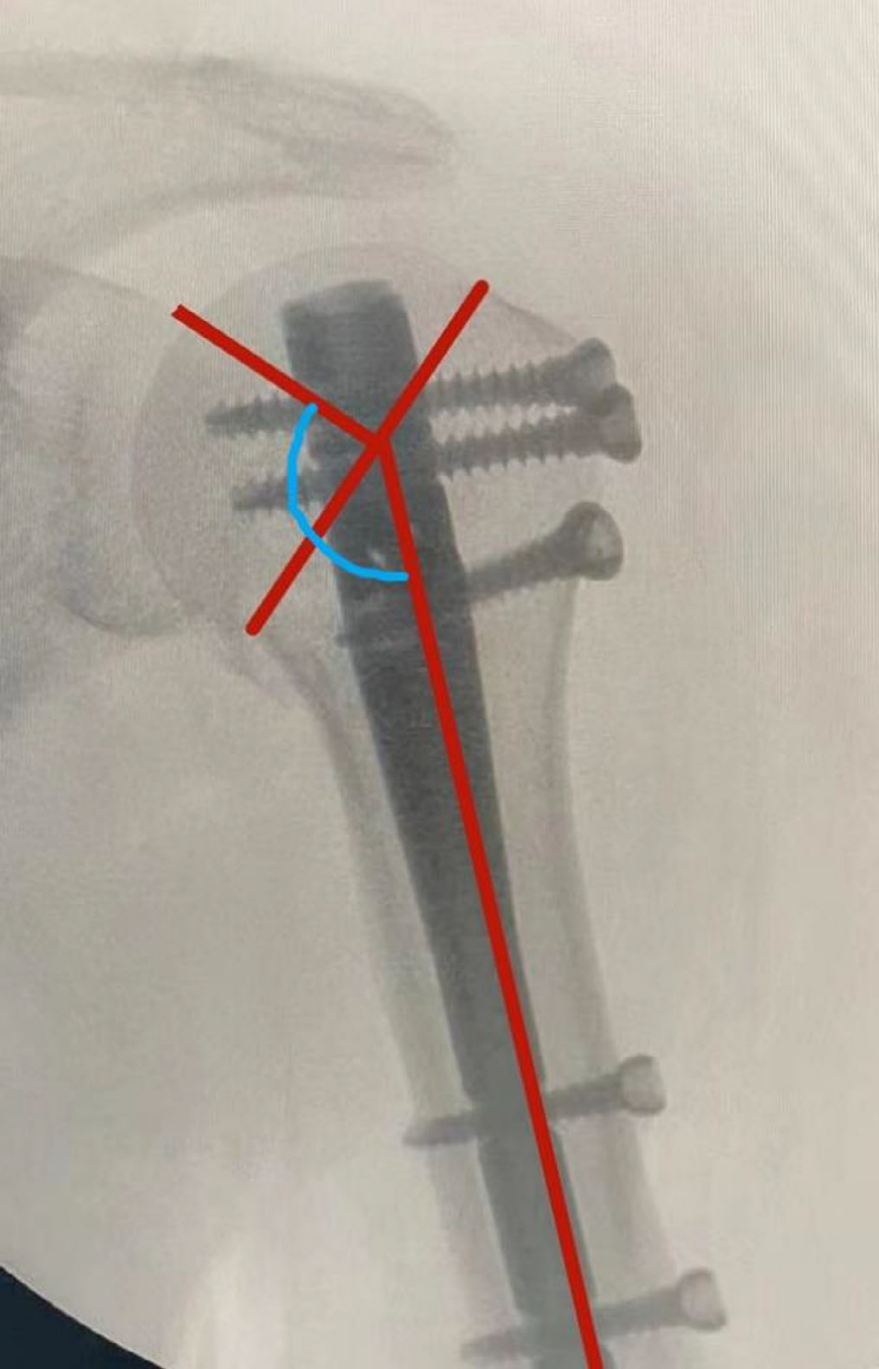
Measurement of neck shaft angle

Preoperative radiographs, including standard anteroposterior (AP) and trans‐scapular (Y) radiographs, three‐dimensional reconstruction computed tomography scans, were used to determine NEER classification of the fracture. And calcar fracture morphology was revealed by two‐dimensional computed tomography scans on coronal plane or three‐dimensional reconstruction computed tomography scans with scapula and clavicle subtraction. Local bone quality in the proximal humerus was assessed by deltoid tuberosity index (DTI), which indicated low local bone quality when lower than 1.4. At the level where outer cortical borders become parallel proximal to the deltoid tuberosity, the ratio between the outer and the inner cortical diameter is calculated^29^ (Figure 3). The assessment of radiographs was done immediate postoperatively, and at final follow‐up. All radiographs were taken by the same two technicians in the same settings in order to minimize potential errors. Also, the radiographs were analyzed by one author. Radiographic union was determined when bridging callus occurred in 3/4 cortices on radiographs. Neck shaft angle (NSA) was defined as the angle between a line along the humeral shaft axis and a line perpendicular to the anatomical neck (Figure 4). Clinical assessments at follow‐up included range of motion, subjective pain level reported by VAS (0–10), Simple Shoulder Test (SST) score (0–12), and American Shoulder and Elbow Surgeons (ASES) score (0–100). According to the obtained SST and ASES scores, it could be divided into four grades, poor (SST was 0–3, ASES was 0–25), fair (SST was 4–6, ASES was 26–50), good (SST was 7–9, ASES was 51–75), and excellent (SST was 10–12, ASES was 76–100). Complications were assessed, including varus malunion, humeral head necrosis, non‐union, infection, and clinical signs and symptoms due to rotator cuff tendon tear or subacromial impingement.

### Statistical Analysis

All analyses were performed with SPSS version 20.0 (IBM Corp., Armonk, NY, USA). Descriptive statistics, including means, standard deviations, were performed for each studied group. To assess differences, a Student t‐test was used for continuous variables and χ^2^ analysis for categorical variables. Univariate logistic regression analysis was used to evaluate the correlation between possible risk factors and loss of NSA. The significance level was set at α = 0.05.

## Results

Eighty‐three patients met the inclusion criteria and were involved in our study who had complete follow‐up and clinical assessment. There were 54 women and 29 men. The average follow‐up was 12.8 months, from 10 to 33 months. The mean age at the time of surgery was 58.6 years, from 20 to 89 years. According to the NEER classification, fractures included 42 two‐part, 37 three‐part, and four four‐part cases. Calcar fractured on both α and β planes in 19 patients, which was defined as comminuted calcar (Table 1). DTI had an average of 1.50 ± 0.19 (range 1.13–2.04).

**TABLE 1:** NEER classification and calcar integrity

	Neer parts			Total
	2	3	4	
Calcar				
Intact	31	31	2	64
Comminuted	11	6	2	19
Total	42	37	4	83

### Radiographic Results

Average immediate postoperative NSA was 145° ± 6° (range, 128°–159°), and NSA at last follow up was 141° ± 6° (range, 125°–156°) (Table 2). The mean loss of NSA was 4° (range, 0°–12°). Only three patients showed a total loss of reduction of greater than 10°. No case with varus malalignment in our study occurred, whose immediate postoperative NSA was less than 120°. The average loss of NSA was 3.84° ± 2.12° in Group A and 4.53° ± 2.95° in Group B. No significant difference was found between two groups (*p* = 0.27). Univariate regression analysis revealed that age, gender, DTI, calcar integrity, or immediate postoperative NSA did not have significant correlation with the degree of NSA loss.

**TABLE 2:** Characteristics and outcomes

	Calcar	Intact	Comminuted	*p* value
	Total (83)	Group A (64)	Group B (19)	
Sex (M/F)	29/54	24/40	5/14	
Age (yr)	58.63 ± 16.30	57.06 ± 16.57	63.89 ± 14.55	0.11
DTI	1.50 ± 0.19	1.51 ± 0.19	1.50 ± 0.19	0.83
NSA loss	4.00 ± 2.34	3.84 ± 2.12	4.53 ± 2.95	0.27
VAS	1.29 ± 0.96	1.19 ± 0.94	1.63 ± 0.96	0.07
ASES	74.84 ± 8.31	75.44 ± 8.16	72.84 ± 8.70	0.23
SST	8.33 ± 1.99	8.61 ± 1.85	7.37 ± 2.22	0.02
FE	127.23 ± 12.33	129.06 ± 11.91	121.05 ± 11.97	0.01
ABD	123.49 ± 9.56	124.22 ± 9.05	121.05 ± 11.00	0.20

Abbreviations: ABD, abduction (in degrees); ASES, American shoulder and elbow surgeons score; DTI, Deltoid tuberosity index; FE, Forward elevation (in degrees); NSA, Neck shaft angle; SST, Simple shoulder test; VAS, Visual analog scale.

### Clinical Outcomes

The mean range of motions of the affected limb at final follow‐up is shown in Table 2. Compared to patients with comminuted calcar, patients with intact calcar demonstrated greater forward flexion range, which was statistically significant (129.06 ± 11.91 vs. 121.05 ± 11.97, *p* = 0.01), and greater range of abduction, which was not statistically significant (124.22 ± 9.05 vs. 121.05 ± 11.00, *p* = 0.20). The average VAS score was 1.29 ± 0.96, and similar VAS scores were revealed between two groups (1.19 ± 0.94 *vs*. 1.63 ± 0.96, *p* = 0.07). The average SST was 8.33 ± 1.99. Among all patients, functional assessment was graded as fair in 13 patients (15.7%), good in 33 patients (39.8%), and excellent in 37 patients (44.6%). Statistically, significant difference was found in SST scores between two groups, which favored patients in Group A (8.61 ± 1.85 vs. 7.37 ± 2.22, *p* = 0.02). The average ASES was 74.84 ± 8.31, which was graded as fair in four patients (4.8%), good in 26 patients (31.3%), and excellent in 53 patients (63.9%). Patients with intact calcar reported similar ASES scores to those with comminuted calcar (75.44 ± 8.16 vs. 72.84 ± 8.70, *p* = 0.23) (Table 3).

**TABLE 3:** Outcomes based on score category

Outcome category	Intact calcar		Comminuted calcar		Total	
	STT	ASES	STT	ASES	STT	ASES
Fair	6	3	7	1	13	4
Good	26	16	7	10	33	26
Excellent	32	45	5	8	37	53

Abbreviations: ASES, American shoulder and elbow surgeons score; SST, Simple shoulder test.

### Complications and Reoperations

**Fig. 5:**
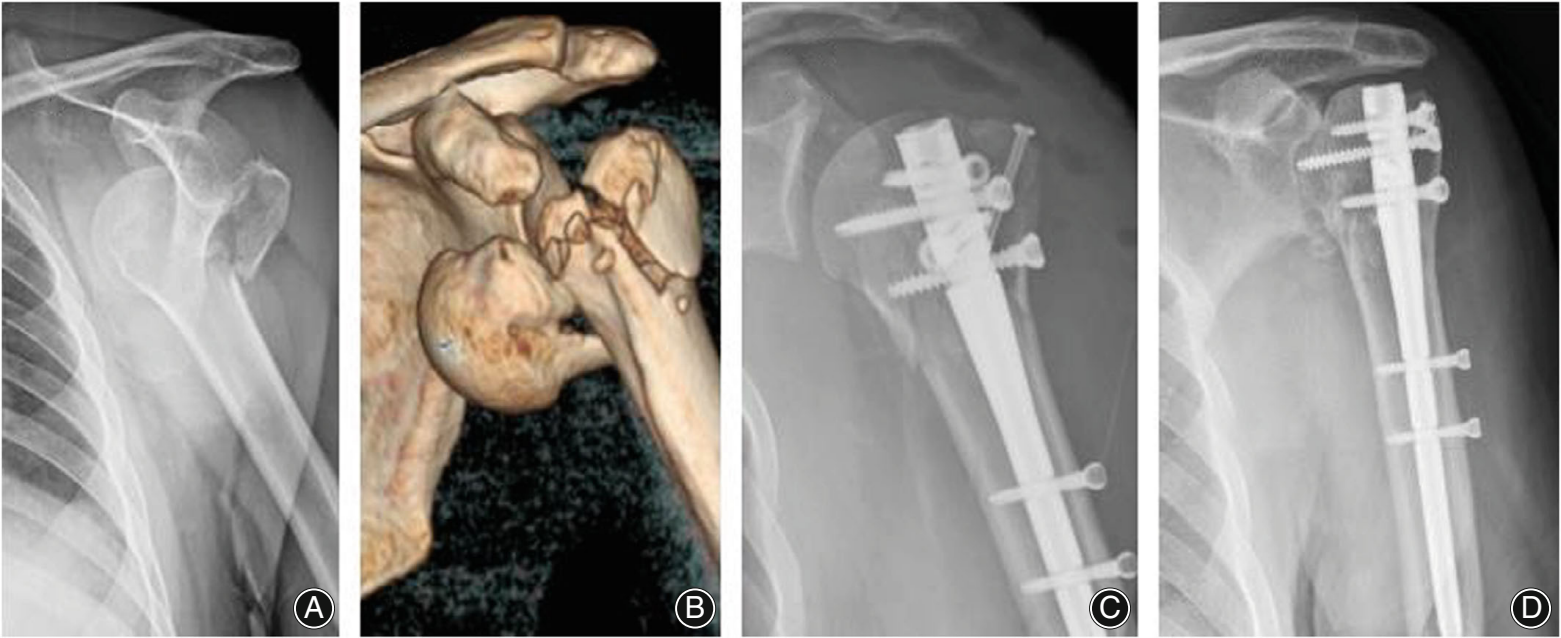
(A) Dislocation and greater tuberosity fracture; (B) Fracture of greater tuberosity and humeral head; (C) Radiograph immediate postoperatively; (D) Radiograph 8 months postoperatively indicating osteonecrosis of the humeral head

One patient (53‐year‐old man, three‐part fracture‐dislocation, intact calcar, DTI = 1.81) demonstrated a proximal interlocking screw cutting through and humeral head necrosis, who received a second surgery for screw removal (Figure 5). No cases of malunion, nonunion, infection, clinical symptom of rotator cuff tear or subacromial impingement occurred during the follow‐up period.

### Typical Cases

**Fig. 6:**
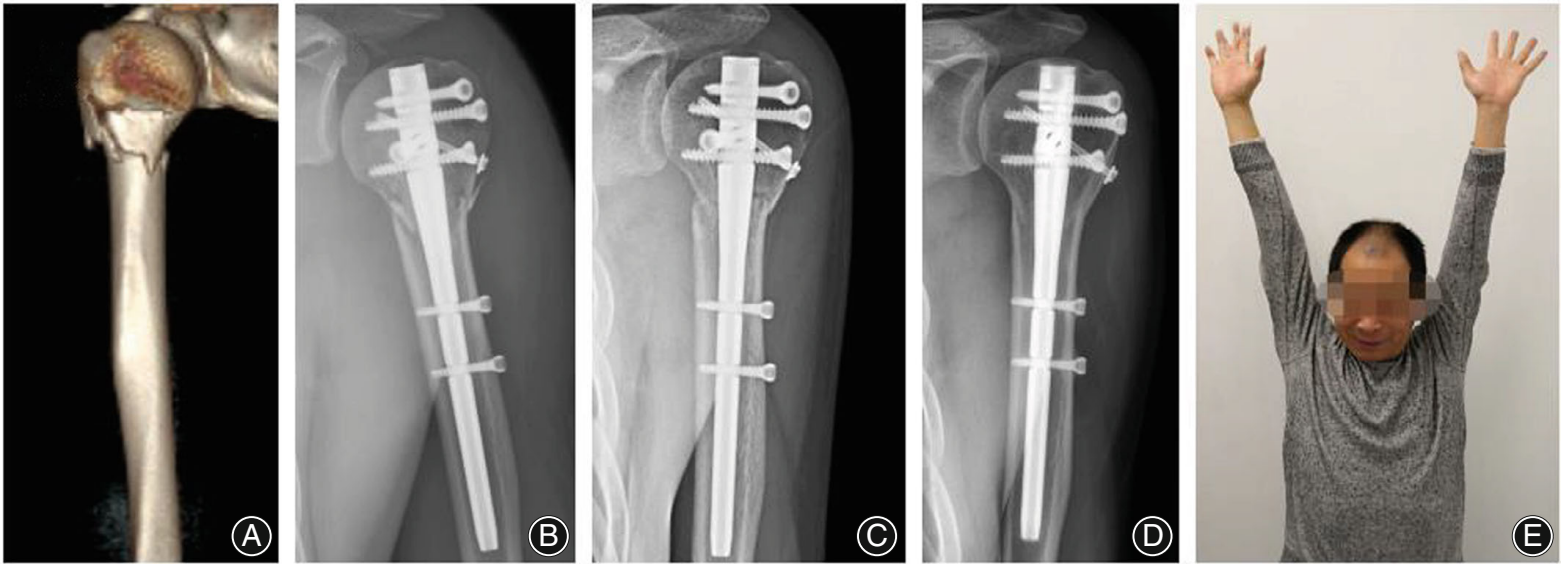
(A) Preoperative 3D‐CT; (B) Radiograph immediate postoperatively; (C) Radiograph 3 months postoperatively; (D) Radiograph 2 years postoperatively; (E) Functional image at final follow‐up

Case 1. A 46‐year‐old man suffered a two‐part proximal humeral fracture with comminuted calcar and was treated with intramedullary nail, whose fracture union was observed 3 months after surgery, with good clinical outcomes at last follow‐up (Figure 6).

**Fig. 7:**
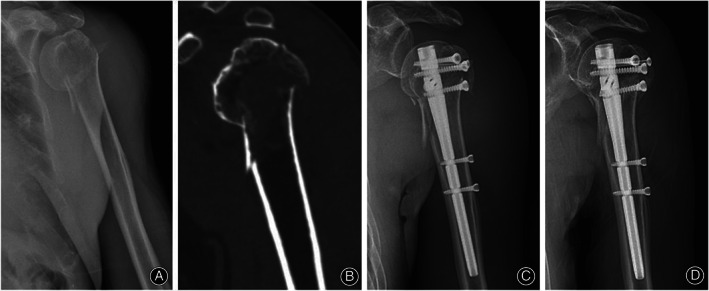
(A) Preoperative radiograph; (B) Preoperative CT scan; (C) Radiograph immediate postoperatively; (D) Radiograph 3 months postoperatively

Case 2. A 76‐year‐old man suffered a varus displaced three‐part proximal humeral fracture with comminuted calcar. Intramedullary nail was used, and fracture union was observed 3 months after surgery (Figure 7).

**Fig. 8:**
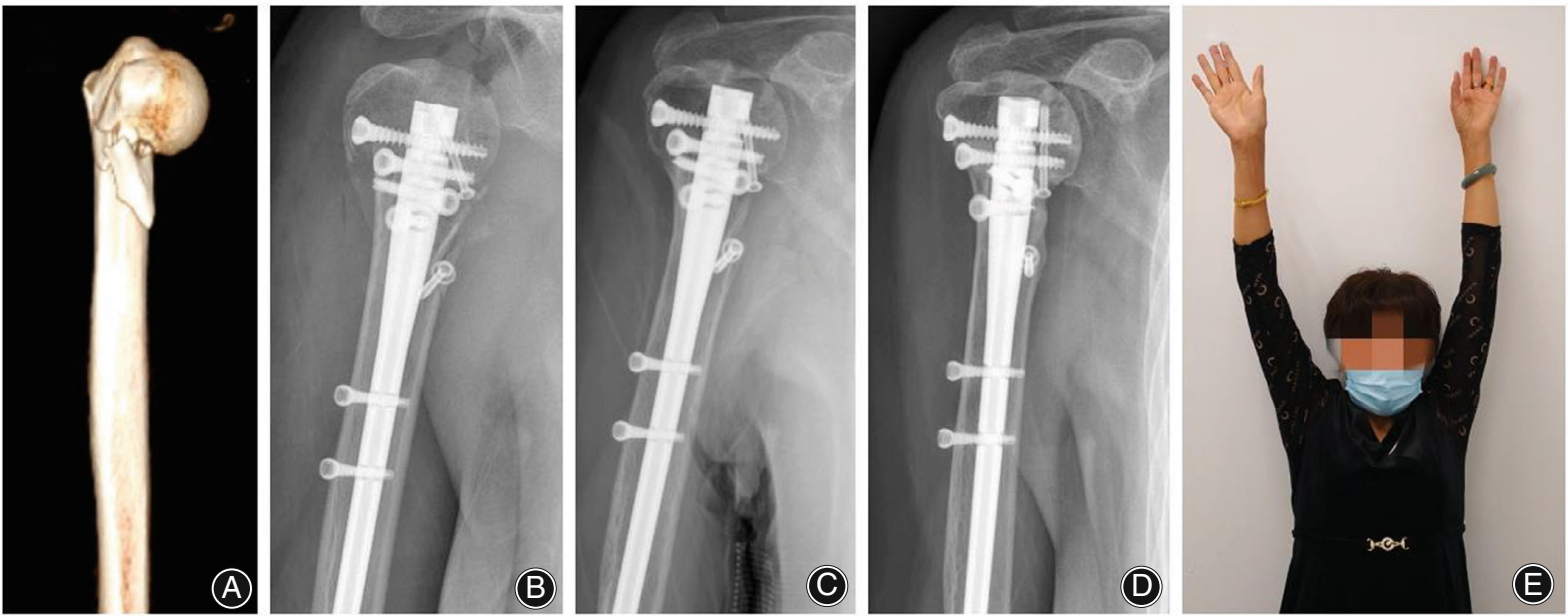
(A) Preoperative 3D‐CT; (B) Radiograph immediate postoperatively; (C) Radiograph 5 months postoperatively; (D) Radiograph 1 year postoperatively; (E) Functional image at final follow‐up

Case 3. A 61‐year‐old woman suffered a varus displaced two‐part proximal humeral fracture with comminuted calcar and was managed with intramedullary nail. Fracture union was observed 5 months after surgery, with good clinical outcomes at last follow‐up (Figure 8).

## DISCUSSION

We found that intramedullary nailing is well suited for displaced proximal humeral fractures, even though the calcar is comminuted, with good early to mid‐term radiographic and functional outcomes and low complication rates.

### Medial Support Importance and Role of Intramedullary Nail

With comminution of the medial calcar, the medial hinge is destroyed, and the varus‐displaced type is the most common unstable fracture of the proximal humerus.^22^ Calcar comminution might imply higher fracture complexity, decreased bone quality, impaired local blood supply, and reduction loss as a result of lack of medial support. Since medial calcar comminution is an important prognostic factor for the clinical outcomes of locking plate fixation for proximal humeral fractures,^26^ its prognostic value is of interest in patients managed with intramedullary nailing. From a biological point of view, intramedullary nails can protect the soft tissue and preserve the blood supply of the humeral head, and its intramedullary fixation offers better biomechanical stability than other fixation techniques.^30^ The third‐generation straight nail used in our study, rather than the curved nail, increases proximal fracture stability due to its more medial entry point, as in the humeral head segment, a safe area is left between the nail insertion hole and the lateral fracture line of the humeral head. Stedtfeld et al.^31^ found that a stable bony ring around the intramedullary nail was necessary to prevent secondary varus displacement.

### Radiographic Evaluation

Wong et al.^32^ reported that secondary loss of reduction with proximal humeral nail was around 10%, but all types of fractures were involved. While only taking two‐part fractures into account, the rate was distinctly lower. In a study of Hatzidakis et al.^33^ they found 2.6% loss of reduction (1/38) with an inappreciable diminution in NSA for the rest cases. Trepat et al.^34^ demonstrated a mean diminution in NSA of 3° in 15 patients. And Zhu et al.^19^ reported no cases of loss of reduction (0/25) in their younger cohort. On the other side, Nolan et al.^35^ found a mean loss of 8° between immediate postoperatively and final follow‐up, and 33% radiographic malunion. Rotman et al.^36^ reported that mean NSA decreased from 139.1° to 122.6° at last follow‐up. Meanwhile, 24% of patients (6/25) presented a NSA diminution more than 20°, and 8% (2/25) ended up with malunion. In this study, on the contrary, no case of malunion or loss of reduction occurred and the mean change in NSA was only 4°, regardless of fracture type and calcar comminution, which reflecting the biomechanical advantages of intramedullary nail. In addition, acceptable initial fracture reduction quality was equally important. Spross et al.,^37^ in their study on proximal humeral plate, showed that the DTI was an important predictor for an acceptable loss of reduction and for maintenance of reduction. However, in this study on intramedullary nail, the DTI was found to have no correlation with NSA change.

### Functional Evaluation

In the study of Osterhoff et al.,^26^ their results showed that medial calcar comminution had a significant impact on the clinical outcome of plate fixation therapy. In their cohort, those with medial calcar comminution had significantly worse functional outcomes and quality of life at follow‐up over 4 years after surgery. Hertel et al.^38^ described predictors of avascular necrosis of the humeral head, finding the importance of medial hinge integrity in particular. However, Osterhoff et al.^26^ found no statistical relationship between calcar comminution and AVN. Therefore, the impression of calcar comminution could not be interpreted solely by the effect on the blood supply to the humeral head and its effect on clinical outcomes. They also found that calcar comminution was relevant to impingement. In our study, patients showed no difference in VAS score, ASES score, and range of abduction between two groups. While, greater range of forward elevation and higher SST score with intact calcar were noted. On the whole, the effect of calcar comminution on clinical outcomes was not equally significant in patients treated with intramedullary nail, as compared to those with plate fixation.

### Strengths and Limitations

The strength of this study was the homogeneity of implant type and treatment performed in the cohort. However, this study had several weaknesses. Major limitations were its retrospective nature and limited sample size; therefore, it was insufficient to properly assess the risk factors for secondary displacement. As the average follow‐up period was about 12 months, we could probably underestimate the occurrence of AVN. While most complications occur during the first year, AVN may occur at longer intervals.^39^ Although we did not find symptoms and signs of rotator cuff tear or subacromial impingement in our patients, it was unclear in our study how many patients suffered an iatrogenic rotator cuff tear, since magnetic resonance imaging or ultrasound was not performed to assess postoperative rotator cuff integrity.

### Conclusions

In our study, intramedullary nail can favorably be used to manage proximal humeral fractures with good early radiographic and functional outcomes, even for those with comminuted calcar. More prospective clinical studies with higher level of evidence are needed to determine the efficacy of intramedullary nailing for the treatment of proximal humeral fractures with comminuted calcar.

#### AUTHOR'S CONTRIBUTION

F. H. drafted the manuscript. W. J. made substantial contributions in the conception of the article. W. X. revised the manuscript. All authors proofread and approved the manuscript.

## CONFLICT OF INTEREST

The authors declare no conflict of interest in regard to the content of this manuscript.
